# Characterization of a single mutation in TraQ in a strain of *Escherichia coli* partially resistant to Qβ infection

**DOI:** 10.3389/fmicb.2015.00124

**Published:** 2015-02-20

**Authors:** Akiko Kashiwagi, Hikari Kitamura, Fumie Sano Tsushima

**Affiliations:** Faculty of Agriculture and Life Science, Hirosaki University, HirosakiJapan

**Keywords:** coevolution, prey–predator, experimental evolution, virulent phage, partial resistance

## Abstract

Bacteria and virulent bacteriophages are in a prey–predator relationship. Experimental models under simplified conditions with the presence of bacteria and bacteriophages have been used to elucidate the mechanisms that have enabled both prey and predator to coexist over long periods. In experimental coevolution conducted with *Escherichia coli* and the virulent RNA bacteriophage Qβ in serial transfer, both coexisted for at least for 54 days, during which time they continued to change genetically and phenotypically. By day 16, an *E*. *coli* strain partially resistant to Qβ appeared and caused an approximately 10^4^-fold decrease in Qβ amplification. Whole-genome analysis of this strain suggested that a single mutation in TraQ was responsible for the partially resistant phenotype. TraQ interacts with propilin, encoded by the *traA* gene and a precursor of pilin, which is a component of the F pilus. The present study was performed to elucidate the mechanism underlying the coexistence of *E. coli* and Qβ by investigating how a mutation in TraQ altered the physiological state of *E. coli*, and thus the amplification of Qβ. Overexpression of wild-type TraQ in the partially resistant *E. coli* strain resulted in recovery of both TraA protein content, including propilin and pilin, and Qβ amplification to levels comparable to those observed in the susceptible strain. Intriguingly, overexpression of the mutant TraQ in the partially resistant strains also increased the levels of TraA protein and Qβ amplification, but these increases were smaller than those observed in the wild-type strain or the partially resistant strain expressing wild-type TraQ. The results of this study represent an example of how *E. coli* can become partially resistant to RNA bacteriophage infection via changes in a protein involved in maturation of a receptor rather than in the receptor itself and of how *E. coli* can stably coexist with virulent RNA bacteriophages.

## INTRODUCTION

There have long been ecological and theoretical investigations regarding why predators do not eradicate their prey (Murdoch and Oaten, 1975; Anderson and May, 1978; Alexander, 1981; Berryman, 1992; Abrams, 2000; Briggs and Hoopes, 2004; Pettorelli et al., 2011; Brockhurst and Koskella, 2013). Bacteria and bacteriophages have been used as model systems of prey–predator interactions to elucidate the fundamental mechanism underlying this issue. Due to their short generation time, large population size, and ease of analyzing phenotypic and genomic changes, theoretical and experimental evolutionary research have been conducted extensively using these systems (Campbell, 1961; Levin et al., 1977; Lenski and Levin, 1985; Lenski, 1988; Schrag and Mittler, 1995; Sasaki, 2000; Paterson et al., 2010; Dennehy, 2012; Brockhurst and Koskella, 2013; Bull et al., 2014). The stable coexistence of bacteria and virulent phages has been widely observed in experimental evolution (Horne, 1970; Chao et al., 1977; Levin et al., 1977; Buckling and Rainey, 2002; Lythgoe and Chao, 2003; Kerr et al., 2006; Kashiwagi and Yomo, 2011; Marston et al., 2012), even though virulent phages kill the host bacteria to release progeny phages.

The existence of refuges for sensitive bacteria or the occurrence of endless arms races were suggested to be necessary to explain the coexistence of bacteria and bacteriophages (Chao et al., 1977; Lenski, 1988; Schrag and Mittler, 1995; Brockhurst et al., 2006). The numerical refuge in which density-dependent protection of susceptible cells from over-predation (Chao et al., 1977), spatial refuges such as wall populations on flasks in continuous culture or solid media used in serial passage (Chao et al., 1977; Lenski, 1988; Schrag and Mittler, 1995), and physiological refuges in which cells become transiently resistant or susceptible have been discussed (Lenski, 1988). In most of these previous studies, DNA bacteriophages, such as T2, T5, T7, λ, and Φ2, were used (Bohannan and Lenski, 2000; Buckling and Rainey, 2002; Dennehy, 2012). Although a great deal of knowledge has been accumulated regarding DNA bacteriophages, little is known about RNA bacteriophages in terms of stable coexistence.

In our previous study, *Escherichia coli* and the lytic RNA bacteriophage Qβ (Qβ) were shown to coevolve for at least 54 days, equivalent to 165 generations, under conditions of serial passage with shaking (Kashiwagi and Yomo, 2011). Qβ is a bacteriophage with a single-stranded RNA genome that specifically infects and lyses *E. coli* cells to release progeny phages (Van Duin and Tsareva, 2006). Phenotypic and genomic analyses indicated the coevolution of both *E. coli* and Qβ. In the course of experimental coevolution, partially resistant *E. coli* appeared in the 54th generation (16th day). Detailed analysis of the partially resistant *E. coli* is necessary to determine how *E. coli* and Qβ coexist in this experimental coevolution system. Genetic analysis revealed a single mutation in the *traQ* gene in the day-16 *E. coli* population (Kashiwagi and Yomo, 2011). It was reported that TraQ protein is a chaperone for insertion of propilin encoded by the *traA* gene (Moore et al., 1982; Kathir and Ippen-Ihler, 1991), and propilin was also reported to be unstable in *traQ*^-^ cells (Maneewannakul et al., 1993). The 13-kDa propilin is processed by peptidase to a 7-kDa pilin and pilin proteins are assembled into filaments (i.e., the F pilus). Qβ adsorbs the F pilus of *E. coli* at the first step of infection (Van Duin and Tsareva, 2006), and the adsorption rate of Qβ on partially resistant cells estimated by first-order kinetics decreased markedly (Kashiwagi and Yomo, 2011). Therefore, the partially resistant phenotype of *E. coli* to Qβ infection observed in coevolution may be correlated with F pilus biosynthesis, especially TraA, and we focused on the relationships among mutation in TraQ, TraA content, and Qβ amplification.

Here, we report that a single amino acid change in TraQ was linked to the reduction of TraA content in the *E. coli* population. In addition, this decrease was recovered by supplying ancestral (wild-type) or mutant-type TraQ from an expression vector, and the ability of Qβ to amplify in the cell also recovered. These results represent one example of how *E. coli* can become partially resistant to RNA bacteriophage infection, which involves changes in a protein related to the maturation of a receptor, in this case the F pilus, rather than changes to the receptor itself. These results suggest that the mutation in TraQ may cause heterogeneity within the *E. coli* population, with a small number of cells supporting the phage population and a large number of cells supporting the *E. coli* population without Qβ infection, even though the *E. coli* cells were genetically identical.

## MATERIALS AND METHODS

### STRAINS, CULTURE MEDIA, AND PLASMID DNA

*Escherichia coli* Anc(C), the partially Qβ infection-resistant mutant strain, M54(C) (Kashiwagi and Yomo, 2011), and DH1 Δ*leuB*::(*gfpuv5-Km^r^*) (hereafter called LKG; Kishimoto et al., 2010) were used to characterize the effects of S21P mutation in TraQ protein and a control F^-^ strain. *E. coli* A/λ (Watanabe et al., 1979) was used as the host strain for titer assay. LB medium (10 g/L tryptone, 5 g/L yeast extract, 10 g/L NaCl; Nakalai Tesque, Kyoto, Japan) was used.

To construct TraQ with a Strep-tag II sequence (Schmidt and Skerra, 2007) at the C-terminus of the ancestral-type TraQ (TraQ_Anctag_) and mutant-type TraQ (TraQ_S21Ptag_), the *traQ* gene was amplified by PCR with Anc(C) and M54(C) genome as the template, the primers traQ_XbaI and traQ_strep_HindIII, and Phusion®; High-Fidelity DNA polymerase (New England Biolabs, Ipswich, MA, USA). The oligonucleotide DNAs used in this study are listed in **Table 1**. The *traQ_Anctag_* and *traQ_S21Ptag_* genes with *Xba*I and *Hin*dIII sites at both ends were subcloned into the *Xba*I/*Hin*dIII sites of pASK-IBA3plus (IBA Biologics GmbH, Goettingen, Germany). The resulting plasmid DNAs were designated as pASK-traQ_Anctag_ and pASK-traQ_S21Ptag_, respectively.

**Table 1:** Oligonucleotide DNA sequence.

Primer name	Sequence (5′→3′)
F_4f_2	ATCAGCGCAATAATTGCCGC
F_4r_2	CGATTATTCCCGTCACGATG
Linker_r	ATTGATGGTGCCTACAG
pACYC_rev2	CCACACATTATACGAGCCG
traA1	GACGAGTGAATTTGGAAAAAAACGACTTCTTTTTGACGGGCGCAGAAGCACCCTGAACAC
traA_f	ATGAATGCTGTTTTAAGTGT
traA_r	TCAGAGGCCAACGACGGCCA
traA_r2	GGCCATACCCACAGCAATAA
traQ_XbaI	TCTAGAAGGAGATATACAATGATAAGTAAACGCAGATT
traQ_strep_HindIII	AAGCTTATTATTTTTCGAACTGCGGGTGGCTCCAGTGAGAGACATGTCCGCCCT
5PpACYC_rev	pTCGGCTCGTATAATGTGTGG
16SrRNA_1	GCTGCCTCCCGTAGGAGT

### DNA SEQUENCING OF THE *traQ* GENE OF ANCESTRAL AND PARTIALLY RESISTANT *E. coli*

To determine the *traQ* gene sequneces of Anc(C) and M54(C), the *traQ* region in 10 colonies each of Anc(C) and M54(C) was amplified by PCR with the primers F_4f_2 and F_4r_2 and Phusion®; High-Fidelity DNA polymerase (New England BioLabs), and PCR products were directly sequenced by the dideoxynucleotide chain termination sequencing method (Sanger et al., 1977).

### ESTIMATION OF Qβ AMPLIFICATION

Anc(C)/pASK-traQ_Anctag_, Anc(C)/pASK-traQ_S21Ptag_, Anc(C)/pASK-IBA3plus, M54(C)/pASK-traQ_Anctag_, M54(C)/pASK-traQ_S21Ptag_, and M54(C)/pASK-IBA3plus were cultured in 5 mL of LB with 100 μg/mL ampicillin overnight and 50 μL of the culture was inoculated into 5 mL of LB with 100 μg/mL ampicillin for approximately 2 h. Aliquots of 1 mL of the culture were transferred into 4 mL of LB medium with 100 μg/mL ampicillin and 100 nM doxycycline-HCl (Dox) and cultured for a further 2 h. Qβ was added to infect the bacterial cells and the free phage was separated immediately or 4 h after infection by centrifugation at 13400 ×*g* for 1 min. The free phage in the supernatant was diluted, the number of plaque forming units per milliliter was determined (PFU/mL), and the amplification ratio was calculated as *x* = N_4_/N_0_, where N_4_ and N_0_ represent 4 h after and initial (0 h) free phage density, respectively. The titer assay was conducted according to the standard method described previously (Carlson, 2005).

### WESTERN BLOTTING ANALYSIS

A polyclonal antibody to TraA protein raised against the keyhole limpet hemocyanin-conjugated peptide (CDLMASGNTTVKATFGKDSS) was obtained from Sigma-Aldrich Japan (Tokyo, Japan). Cell preculture was conducted as described in the Section “Estimation of Qβ Amplification.” For induction with 100 nM Dox, Dox was added to the 2-h culture and cells were cultured for a further 5.75 h. Without Dox induction, the cells were cultured for 7.25 h in LB medium. Proteins from the cells obtained from 0.1 mL of culture with OD_600_ = 2.0 were subjected to SDS-PAGE using Any kD^TM^ Mini-PROTEAN®; TGX^TM^ precast gels (Bio-Rad Laboratories, Hercules, CA, USA), and the TraA protein was determined by Western blotting analysis with anti-TraA antibody and HRP-conjugated goat anti-rabbit polyclonal antibody (Santa Cruz Biotechnology, Inc., Santa Cruz, CA, USA) as the primary and secondary antibodies, respectively, diluted with Can Get Signal®; immunoreaction enhancer solution (Toyobo Co. Ltd., Osaka, Japan). TraQ with a Strep-tag II at the C-terminus was detected using Precision Protein^TM^ Strep-Tactin-HRP conjugate (Bio-Rad Laboratories). The signals were detected with Chemi-Lumi One L (Nakalai Tesque).

### NORTHERN HYBRIDIZATION ANALYSIS

To compare the *traA* mRNA contents by Northern hybridization, Anc(C), M54(C), and LKG with pASK-IBA3plus were cultured in LB medium and total RNA was extracted from the cells in logarithmic phase using the SV Total RNA isolation system (Promega, Madison, WI, USA) according to the manufacturer’s instructions. Total RNA from cells obtained from 0.16 mL of culture with OD_600_ = 0.27 was subjected to Northern hybridization using a digoxigenin-labeled single-stranded DNA probe, i.e., traA1 for *traA* and 16SrRNA_1 for 16SrRNA, as an indicator of the amount of total RNA used (Moran et al., 1995). The signals were detected with CDP-Star (GE Healthcare UK Ltd., Little Chalfont, UK). DynaMarker®; Prestain Marker for RNA High (BioDynamics Laboratory Inc., Tokyo, Japan) was used to obtain the standard curve for RNA length.

### DETERMINATION OF 5′- AND 3′-TERMINAL SEQUENCES OF *traA* mRNA

To determine the 5′-terminal sequence of *traA* mRNA, cDNA was synthesized using total RNA of Anc(C) as the template, SuperScript®; III reverse transcriptase (Life Technologies, Carlsbad, CA, USA), and traA_r primer. The 5′-phosphorylated DNA linker 5PpACYC_rev was ligated at the 3′-terminus of the first strand cDNA with T4 RNA ligase 1 (New England Biolabs). PCR was performed using the resultant cDNA as the template, PrimeSTAR®; HS DNA polymerase (Takara Bio Inc., Shiga, Japan), and the primers pACYC_rev2 and traA_r2. To determine the 3′-terminal sequence of *traA* mRNA, universal miRNA cloning linker (5′-rAppCTGTAGGCACCATCAAT-NH2-3′; New England Biolabs) was ligated with the 3′-terminus of total RNA of Anc(C) using T4 RNA ligase 1 (New England Biolabs). The first strand cDNA was synthesized using the primer Linker_r and SuperScript®; III reverse transcriptase (Life Technologies), and then purified cDNA was subjected to PCR using the primers Linker_r and traA_f. Three and two bands of PCR products for 5′- and 3′-terminal sequence determination, respectively, were sliced from the gel and subcloned using a Zero Blunt®; TOPO®; PCR Cloning Kit for Sequencing (Life Technologies). Three to six clones were randomly picked and sequenced by the dideoxynucleotide chain termination sequencing method.

### STATISTICAL ANALYSIS

Amplification ratios were compared by one-way ANOVA with the *post hoc* Tukey’s test (Zar, 2010). In all analyses, values of log_10_ (N_4_/N_0_) of each strain were used for statistical analysis. The Studentized range, *q*, is shown in the text. In all analyses, *P* < 0.01 was taken to indicate statistical significance.

## RESULTS

### RECOVERY OF Qβ AMPLIFICATION IN RESISTANT *E. coli* BY SUPPLYING TraQ

We first analyzed the *traQ* gene sequences from 10 single colonies derived from the coevolved *E. coli* population to confirm that the majority harbored the T61C mutation. We picked 10 single colonies from each of Anc(C) and M54(C) populations, which were the initial and day-16 *E. coli* populations in the coevolution experiment (Kashiwagi and Yomo, 2011). All 10 colonies of Anc(C) had T and all 10 colonies of M54(C) had C at position 61, and this T61C mutation resulted in S21P in the TraQ protein.

**FIGURE 1:**
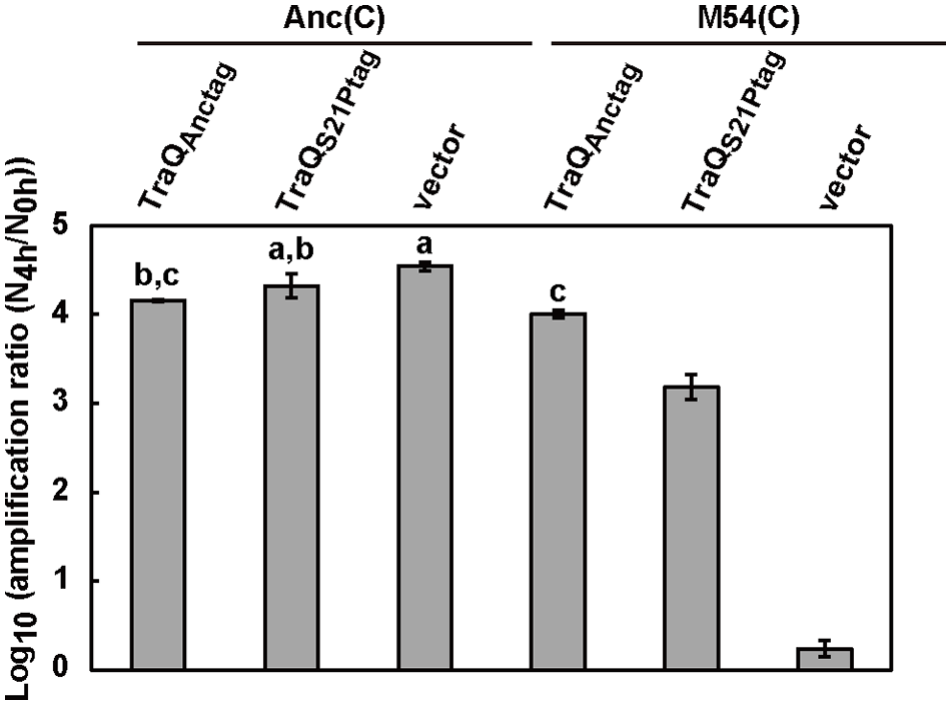
**Amplification ratio of Qβ on each strain.** The amplification ratios of Qβ were measured as the increase of free phage after 4 h of infection on Anc(C) and M54(C) harboring three different plasmids, i.e., pASK-traQ_Anctag_, pASK-traQ_S21Ptag_, and vector (pASK-IBA3plus). Data are expressed as averages ±SD (*n* = 3). Bars with identical letters are not significantly different from each other (Tukey’s test, *P* < 0.01).

We analyzed the amplification ratio of Qβ on Anc(C) and M54(C) that harbored only the vector (pASK-IBA3plus) to determine the extent of reduction in the amplification ratio of Qβ on M54(C). Anc(C) and M54(C) expressed inherent ancestral-type TraQ protein and mutant-type TraQ protein from the F plasmid, respectively. The amplification ratio of Qβ was calculated as described in the Section “Materials and Methods.” The amplification ratios of Qβ on Anc(C)/pASK-IBA3plus and M54(C)/pASK-IBA3plus were 3.5 × 10^4^ and 1.8, respectively (**Figure 1**). Although the amplification ratio on M54(C) was much lower than that on Anc(C), Qβ could undergo amplification on M54(C), indicating that M54(C) had a partially rather than fully resistant phenotype.

To analyze whether ancestral-type TraQ expression in M54(C) could compensate for Qβ amplification in this strain, ancestral-type TraQ with the Strep-tag II sequence at the C-terminus was expressed from the P_tetA_ promoter by doxycycline (Dox) induction. We designated ancestral and mutant-type TraQ with Strep-tag II sequence at the C-terminus expressed from the expression vector as TraQ_Anctag_ and TraQ_S21Ptag_ to allow them to be distinguished from the inherent TraQ_Anc_ and TraQ_S21P_ derived from the F plasmid, respectively. First, to examine whether TraQ_Anctag_ overexpression altered the amplification ratio of Qβ on Anc(C), we compared the amplification ratios of Qβ on Anc(C)/pASK-traQ_Anctag_ and Anc(C)/pASK-IBA3plus under conditions of Dox induction. The amplification ratio of Qβ on Anc(C) overexpressing TraQ_Anctag_ was 1.4 × 10^4^, which was lower than that on Anc(C) harboring only the vector, 3.5 × 10^4^, suggesting that overexpression of TraQ may be slightly deleterious for Qβ amplification (one-way ANOVA *F_5,12_* = 920, *P* < 0.01; *post hoc* Tukey’s test *q* = 7.3, *P* < 0.01; **Figure 1**). Second, the amplification ratio of Qβ on M54(C) overexpressing TraQ_Anctag_ was 1.0 × 10^4^, which was greater than the value of 1.8 on M54(C) carrying only the vector (one-way ANOVA *F*_5,12_ = 920, *P* < 0.01; *post hoc* Tukey’s test *q* = 70.5, *P* < 0.01), and the amplification ratio of M54(C) overexpressing TraQ_Anctag_ was comparable to that of Anc(C)/pASK-TraQ_Anctag_ (one-way ANOVA *F*_5,12_ = 920, *P* < 0.01; *post hoc* Tukey’s test *q* = 2.76, *P* = 0.42; **Figure 1**). These results showed that the amplification ratio of Qβ on M54(C) was recovered by supplying TraQ_Anctag_. Intriguingly, supplying mutant-type TraQ, TraQ_S21Ptag_, to M54(C) also rescued the amplification of Qβ on this strain. When TraQ_S21Ptag_ was overexpressed by Dox induction in M54(C), the amplification ratio of Qβ on the strain was 1.6 × 10^3^, which was greater than that of M54(C) with the vector alone (one-way ANOVA *F*_5,12_ = 920, *P* < 0.01; *post hoc* Tukey’s test *q* = 55.1, *P* < 0.01), but was lower than that of M54(C) overexpressing TraQ_Anctag_ (one-way ANOVA *F*_5,12_ = 920, *P* < 0.01; *post hoc* Tukey’s test *q* = 15.4, *P* < 0.01; **Figure 1**). These results indicated that overexpression of mutant-type TraQ in M54(C) could partially, but not completely, compensate for the decrease in amplification of Qβ on the partially resistant cells.

### RECOVERY OF Qβ AMPLIFICATION LINKED TO AN INCREASE IN TraA

**FIGURE 2:**
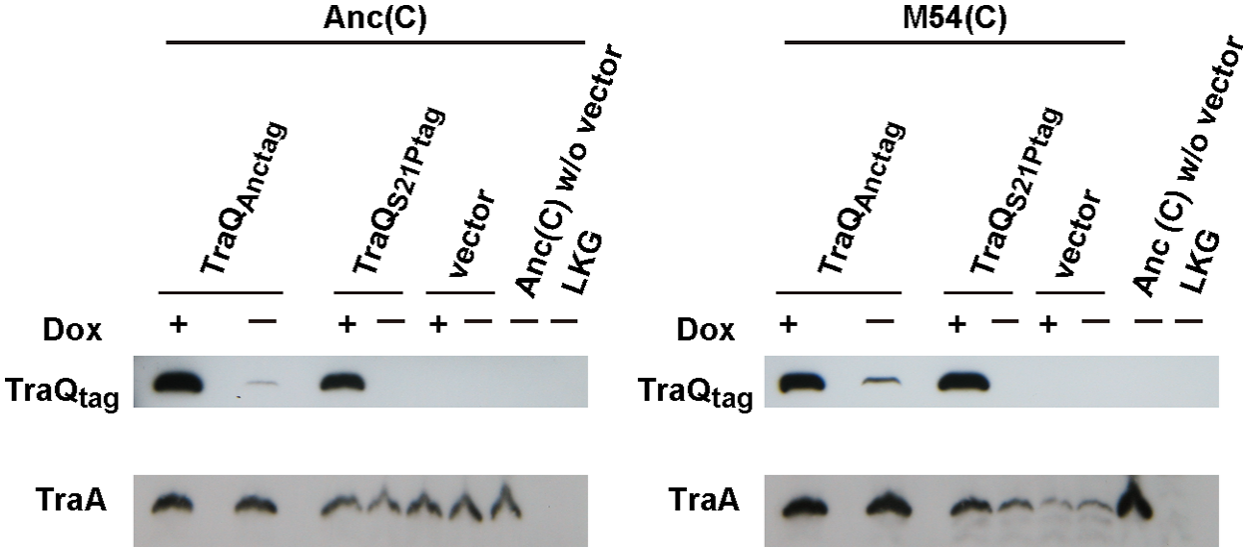
**TraA expression analysis.** The TraA and TraQ with Strep-tag II sequence contents of Anc(C) and M54(C) harboring three different plasmids, i.e., pASK-traQ_Anctag_, pASK-traQ_S21Ptag_, and vector (pASK-IBA3plus), were analyzed. Anc(C) without vector and LKG were used as control strains that expressed and did not express TraA, respectively. (+) and (-) represent with and without 100 nM Dox, respectively. Top and bottom represent Strep-Tactin detection for TraQ with Strep-tag II sequence at the C-terminus and anti-TraA detection for TraA.

We analyzed the TraA content by Western blotting to investigate the links between the mutation in TraQ and TraA content. Proteins derived from the same cell numbers calculated by the values of OD_600_ were subjected to SDS-PAGE. When we compared the levels of TraA produced by Anc(C) and M54(C) with vector (pASK-IBA3plus) and Anc(C) without the vector, the signal strength of TraA of Anc(C) with vector was almost the same as that of Anc(C) without the vector independent of Dox induction (**Figure 2**), but the TraA content of M54(C) with vector was extremely low independent of Dox induction (**Figure 2**). As no signal was detected in the lane for the F^-^ control strain, LKG, M54(C) had not entirely lost TraA. These results showed that M54(C) had markedly decreased propilin and/or pilin content.

The expression levels of ancestral and mutant-type TraQ with the Strep-tag II sequence at its C-terminus were determined with Strep-Tactin. When TraQ_Anctag_ was expressed in M54(C), the signal level for TraA was almost the same as that of Anc(C; **Figure 2**). In this case, the TraA contents were independent of Dox induction. Next, we supplied TraQ_S21Ptag_ to M54(C) with and without Dox induction. The TraA content in M54(C) increased with Dox induction, but the level was lower than that in M54(C) supplied with TraQ_Anctag_ (**Figure 2**). The TraA content of M54(C) also increased without Dox induction, but the strength of the signal was smaller than that under conditions of Dox induction. With supply of TraQ_S21Ptag_, the TraA content was dependent on the expression level of TraQ_S21Ptag_. These observations indicated that a small amount of ancestral-type TraQ in the partially resistant cells was sufficient to recover the TraA content, and a large amount of mutant-type TraQ protein increased the TraA content but the level was lower than that of the ancestral cells. Therefore, these results showed that a single mutation in TraQ resulted in a decrease of TraA content in the partially resistant cells.

### *traA* mRNA EXPRESSION LEVEL IN M54(C)

**FIGURE 3:**
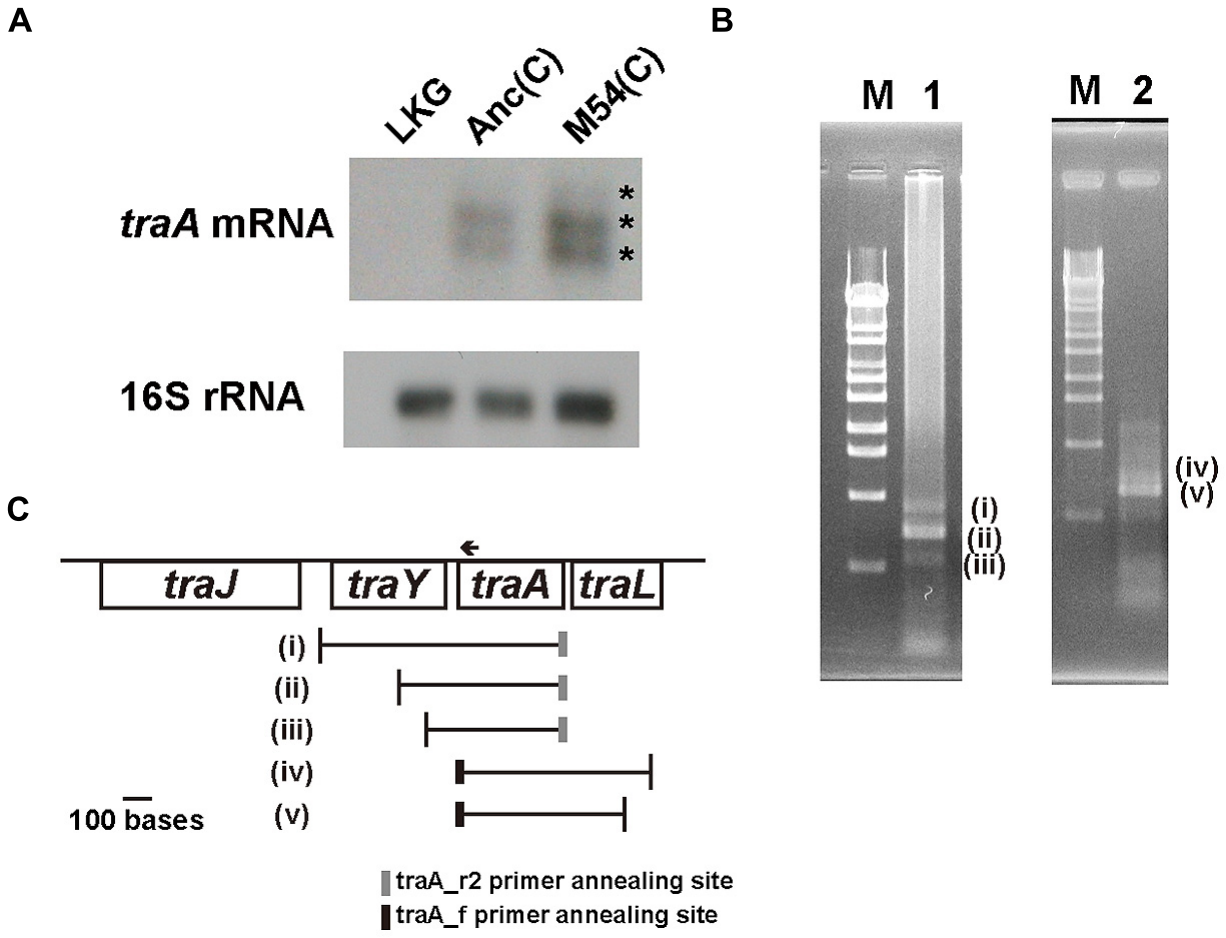
**mRNA of *traA* gene analysis. (A)** Northern hybridization for *traA* mRNA and 16S rRNA using the total RNA of LKG/pASK-IBA3plus; left, Anc(C)/pASK-IBA3plus; middle, M54(C)/pASK-IBA3plus; right. The upper and lower figures are X-ray films of *traA* mRNA and 16S rRNA, respectively. The asterisks (*) indicate the three signals (one weak and two strong). **(B)** RT-PCR for Anc(C)/pASK-IBA3plus to determine the 5′- and 3′-terminal sequences of *traA* mRNA. Lambda DNA digested with *Sty*I was used as a molecular size marker; lane M. RT-PCR products for determination of the 5′-terminus. The three bands were designated as (i), (ii), and (iii), respectively; lane 1. RT-PCR products for determination of the 3′-terminus. The two bands were designated as (iv) and (v), respectively; lane 2. **(C)** Schematic representations of the start and end positions of *traA* mRNA. The vertical lines represent the positions of 5′- and 3′-terminal sequences of (i)–(v) shown in **(B)**. The gray and black boxes represent the positions of traA_r2 and traA_f primer annealing sites in RT-PCR. The arrow represents the position of the traA1 probe annealing site for Northern hybridization.

To determine whether the decrease of TraA protein content in the partially resistant cells was due to a decrease in *traA* mRNA content, we analyzed the *traA* mRNA level by Northern hybridization. No signal was detected in the lane for the F^-^ control strain LKG (**Figure 3A**), while signals were observed in the lanes for the Anc(C) and M54(C) strains (**Figure 3A**). The difference in signal strength of *traA* mRNA between Anc(C) and M54(C) may have been due to differences in the amount of total RNA loaded per lane, which was determined based on the 16SrRNA signal strength, although equal amounts of total RNA were loaded in each lane as calculated from the OD_600_. Therefore, we assumed that the mRNA levels were the almost the same for Anc(C) and M54(C).

As shown in **Figure 3A**, three bands were observed on Northern hybridization; one was weak and two were strong. The three bands corresponded to approximately 800, 610, and 460 bases, respectivel y, calculated using the standard curve obtained with the molecular weight markers. As the *traA* gene is 366 bases in length, the mRNA may contain upstream and/or downstream sequences. To determine the 5′- and 3′-terminal sequences of *traA* mRNA, RT-PCR was performed as described in the Section “Materials and Methods.” For 5′-terminal analysis of mRNA, we added a linker to the 3′-terminus of the first strand cDNA and conducted PCR. At least three PCR products, two of which were clear and the remaining one was weak, were obtained (**Figure 3B**, left). For 3′-terminal analysis of mRNA, we added a linker at the 3′-terminus of the mRNA and conducted RT-PCR. At least two PCR products were obtained, one of which was clearly observed and the other was weak (**Figure 3B**, right). The sequences of these PCR products, designated as (i)–(v), were analyzed and the starting and termination positions of the mRNA including *traA* were determined (**Figure 3C**). The starting and terminating positions described below are numbered according to GenBank accession number AP001918.1. Analysis of six clones of (i) showed that mRNAs started from a position upstream of the *traY* gene; five started at position 67808 and one started at position 67754. Analysis of six clones of (ii) showed that it started from within the *traY* gene; four started at position 68000 and two started at position 67999. Analysis of five clones of (iii) showed that mRNAs started from within the *traY* gene; one started at position 68165, two at 68166, one at 68167, and one at 68189 (**Figure 3C**). Analysis of five clones of (v) showed that mRNAs terminated within the *traL* gene; four terminated at 68818 and one at 68819. Analysis of three clones of (iv) also showed that the mRNAs terminated within the *traL* gene; all three terminated at position 68902. These results indicated that the total RNA included mRNAs encoding *traA* of various lengths with different upstream and downstream sequences. Therefore, all three bands observed on Northern hybridization should contain the *traA* gene sequence.

## DISCUSSION

We reported previously that *E. coli* and Qβ coexisted in serial passage and both continued to change genetically and phenotypically (Kashiwagi and Yomo, 2011). Here, we characterized partially resistant *E. coli* obtained in the previous study and demonstrated links among TraQ content, TraA content, and amplification of Qβ.

In this study, overexpression of mutant-type TraQ_S21Ptag_ was shown to result in an increase in TraA content and recovery of Qβ amplification. These observations indicated that TraQ_S21P_ had not entirely lost its function. The S21P mutation may alter the activity of TraQ, such as changing the binding affinity between TraQ and TraA, and may result in a decrease of TraA content in the M54(C) population and reduce the possibility of Qβ infection by decreasing either the number of cells with F pili or the amount of F pili in each cell in the population, as TraQ protein binds propilin that is a precursor of mature TraA and the first 21 amino acids are important for this binding (Harris et al., 1999). In this study, not only TraQ_Anctag_ but also the TraQ_S21Ptag_ increased the TraA contents of M54(C) without Dox induction. The copy numbers of pASK-TraQ_S21Ptag_, which has the ColE1 replication origin, and F plasmid in the cell are 15–20 and 1, respectively (Snyder and Champness, 2007). Therefore, introduction of pASK-TraQ_S21Ptag_ into the cell increased the copy number of the *traQ* (T61C) gene and therefore may have increased the concentration of mutant-type TraQ in the cell.

It has been reported that F^+^ cells in *E. coli* populations are heterogeneous in the number of F pili per cell and in the length of F pili through the cycles of extension and retraction (Clarke et al., 2008; Silverman and Clarke, 2010). In addition, it is widely accepted that even *E. coli* with the same genotype show phenotypic diversity due to the stochasticity in living organisms (Elowitz et al., 2002; Kashiwagi et al., 2006; Bressloff, 2014). Therefore, even in Anc(C), the population would be heterogeneous in both number and length of F pili per cell. The single mutation of TraQ would decrease the percentage of cells that could be infected by Qβ in the population, even though the M54(C) population was genetically identical throughout the community. The mutation was introduced into the region involved in binding with propilin (Harris et al., 1999) and protein–protein binding is one of the stochastic processes in a cell because it is a collision reaction and reducing numbers of interacting molecules in a cell would increase the fluctuation in number of bound proteins (Bressloff, 2014). Therefore, there would be at least three types of players in the community: a small proportion of cells supporting the phage population, a large proportion of cells supporting the *E. coli* population due to escape from Qβ infection, and Qβ itself. This heterogeneity would result in the partially resistant phenotype of the M54(C) population, as we assessed the phenotype based on the amplification ratio of Qβ in the population and not in single infected cells. As we evaluated the TraA and TraQ contents and Qβ amplification of the population and not of single cells, there are at least two plausible explanations for the partial resistance. The first is that every cell had low levels of TraA or F pili, and the second is that only a small portion of cells in the population had sufficient F pili for Qβ adsorption. In both cases, at least two types of cell—a minor population infected by Qβ and a major population that was not infected by Qβ—may emerge from *E coli* with the identical genotype.

The physiological refuge hypothesis has been reported as one of the mechanisms allowing the coexistence of bacteria and bacteriophages by providing phenotypic heterogeneity in resistance within the bacterial population (Lenski, 1988; Schrag and Mittler, 1995). The results of the present study suggested that the coevolved *E. coli* in experimental evolution would generate phenotypic heterogeneity with both resistant and susceptible cells, as suggested by the physiological refuge hypothesis.

Many resistance mechanisms of bacteria for DNA bacteriophages have been reported, such as preventing phage adsorption, preventing phage DNA entry, cutting phage nucleic acids, abortive infection, and phase variation (Hancock and Reeves, 1975; Labrie et al., 2010; Bikard and Marraffini, 2012). In preventing phage adsorption, surface receptors of bacteria for phage infection were modified, masked by proteins, or blocked by exopolysaccharide (Labrie et al., 2010). However, there have been few discussions regarding the mechanisms of resistance to RNA bacteriophages. Here, we first reported one of the mechanisms underlying partial resistance of *E. coli* to the RNA bacteriophage Qβ that would be included in preventing phage adsorption: a decrease in chance of phage adsorption by reducing the receptor contents in the host population by changing a single amino acid on the protein related to production of the mature receptor (F pilus), not the receptor itself. In addition, the results of this study also suggested that the phenotypic fluctuation caused by changing a single amino acid on the protein would facilitate long-term coexistence of both predator (Qβ phage) and prey (*E. coli*).

## AUTHOR CONTRIBUTIONS

AK designed the research. AK, HK, and FST carried out the experiments and analyzed the data. AK wrote the manuscript.

## Conflict of Interest Statement

The authors declare that the research was conducted in the absence of any commercial or financial relationships that could be construed as a potential conflict of interest.
